# Availability of Real-World Data in Italy: A Tool to Navigate Regional Healthcare Utilization Databases

**DOI:** 10.3390/ijerph17010008

**Published:** 2019-12-18

**Authors:** Edlira Skrami, Flavia Carle, Simona Villani, Paola Borrelli, Antonella Zambon, Giovanni Corrao, Paolo Trerotoli, Vincenzo Guardabasso, Rosaria Gesuita

**Affiliations:** 1 Centre of Epidemiology, Biostatistics and Information Technology, Università Politecnica delle Marche, 60126 Ancona (AN), Italy; e.skrami@staff.univpm.it (E.S.); r.gesuita@staff.univpm.it (R.G.); 2 Department of Public Health, Experimental and Forensic Medicine, University of Pavia, 27100 Pavia (PV), Italy; simona.villani@unipv.it (S.V.); paola.borrelli@unipv.it (P.B.); 3 Department of Statistics and Quantitative Methods, University of Milano-Bicocca, 20126 Milano (MI), Italy; antonella.zambon@unimib.it (A.Z.); giovanni.corrao@unimib.it (G.C.); 4 Department of Biomedical Sciences and Human Oncology, University of Bari, 70121 Bari (BA), Italy; paolo.trerotoli@uniba.it; 5 Teaching Hospital “Policlinico-Vittorio Emanuele”, University of Catania, 95123 Catania (CT), Italy; guardabasso@policlinico.unict.it

**Keywords:** healthcare utilization databases, population based, epidemiology, Italy

## Abstract

The purpose of the study was to map and describe the healthcare utilization databases (HUDs) available in Italy’s 19 regions and two autonomous provinces and develop a tool to navigate through them. A census of the HUDs covering the population of a single region/province and recording local-level data was conducted between January 2014 and October 2016. The characteristics of each HUD regarding the start year, data type and completeness, data management system (DMS), data protection procedures, and data quality control adopted were collected through interviews with the database managers using a standard questionnaire or directly from the website of the regional body managing them. Overall, 352 HUDs met the study criteria. The DMSs, anonymization procedures of personal identification data, and frequency of data quality control were fairly homogeneous within regions, whereas the number of HUDs, data availability, type of identification code, and anonymization procedures were considerably heterogeneous across regions. The study provides an updated inventory of the available regional HUDs in Italy and highlights the need for greater homogeneity across regions to improve comparability of health data from secondary sources. It could represent a reference model for other countries to provide information on the available HUDs and their features, enhancing epidemiological studies across countries.

## 1. Introduction

In the past few decades, large amounts of information underwent digitalization in healthcare, where a number of administrative data related to the utilization of healthcare services and financial and clinical information are routinely and continuously collected in large databases (healthcare utilization databases, HUDs). In Italy, the National Health Service (NHS) provides healthcare to all residents (about 60 million), irrespective of income, gender, or other factors. Healthcare is publicly financed, and services are either free at the point of delivery or involve co-payment of a small flat rate [1]. The Italian NHS is decentralized and organized at three levels: national, regional (19 regions and two autonomous provinces), and local. The general NHS objectives and principles are set at the national level, which also allocates the financial resources. Regional authorities are responsible for organizing and managing the healthcare services, which are delivered through local structures, as well as public and private accredited providers (local facilities providing healthcare services on behalf of the NHS) [2].

Data on the services provided to residents are collected by hospitals and local healthcare structures, entered into structured data files (HUDs) by dedicated regional offices, and periodically sent to the Ministry of Health. Data are registered and stored according to the Italian and European General Data Protection Regulation [3,4]. HUDs are then used at the regional and national level for purposes such as reimbursements, health expenditure monitoring and control, and healthcare service performance assessments. In particular, hospitals and local healthcare structures (i) provide healthcare services supporting the relative costs, (ii) register information related to the services and costs, and (iii) periodically send data to dedicated offices of their own region in order to receive the reimbursement of the provided healthcare services. Reimbursements require high data completeness and quality. Although a few mandatory HUDs were set up in the early 1990s [5], the vast majority were established in 2003 [6] or later.

As a result of NHS decentralization, the mandatory HUDs were set up at different times. The regions/provinces also set up other electronic databases, such as disease and mortality registries, not for administrative use but to monitor the health status of the population.

Electronic databases are valuable data sources for epidemiological studies [7,8,9,10,11], since they can be used for a variety of purposes such as to help estimate the incidence and prevalence of chronic and acute conditions, conduct pharmaco-epidemiological investigations, identify health determinants, assess healthcare service performance [12,13,14,15,16,17], and perform health technology assessments [18,19].

Although a number of studies drew data from HUDs to produce scientific evidence, very few did so by combining HUDs from different Italian regions. The reasons probably include difficulties in HUD accessibility, heterogeneous information systems, and inconsistent data completeness and quality across databases. The ability to combine regional HUDs would not only provide national-level data, but also enable comparing disease burden and healthcare service performance and expenditure among regions; it would also allow exploring rare diseases and their determinants, the adverse events of treatments, and the effects of innovative therapies. A tool enabling easy retrieval of the information stored in HUDs spanning a discrete geographical area and population would enhance the use of real-world data in epidemiological and clinical research.

The aims of this study were to make an inventory of the Italian regional HUDs, to describe them in terms of start year, data type and completeness, data management system (DMS), quality control strategy, and data protection procedures in place, and to develop a tool to navigate through them.

## 2. Materials and Methods 

From January 2014 to October 2016, a survey aimed at identifying the HUDs active in Italy’s 19 regions and two autonomous provinces (Trento and Bolzano) was performed by the Italian Society of Medical Statistic and Clinical Epidemiology’s Working Group on Observational Studies. The survey was funded by the Italian Ministry of Health (RF-2010-2315604) in the framework of the research project ARCHES, “Electronic health databases as a source of reliable information for effective health policy” [20]. The HUDs were included in the survey according to the following inclusion criteria: covering the whole population of a single region and recording local-level data in which the observation unit is the healthcare service.

**Figure 1:**
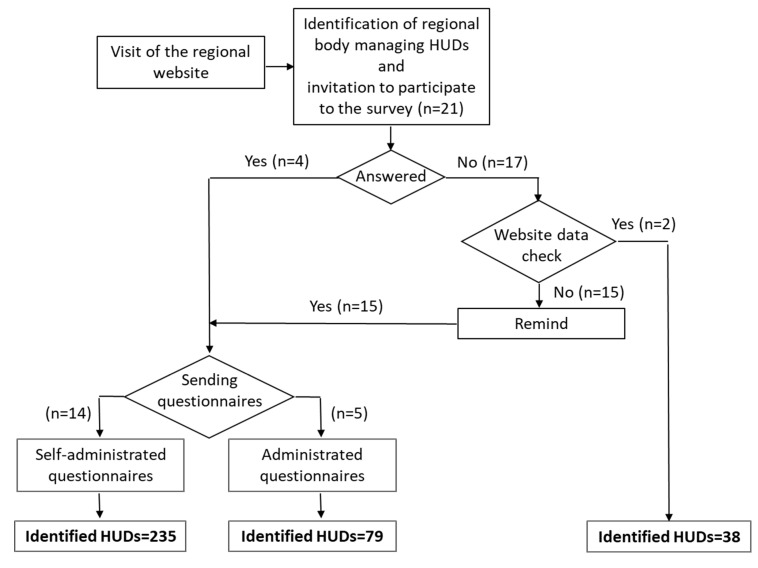
Flow chart of the survey process.

The regional bodies managing HUDs were identified by the website of the institution and invited to participate. For those answering the invitation, a standard questionnaire (Table S1, Supplementary Materials) was sent (a user guide was also provided) that was either self-administrated or administrated by one of the authors. In case of no answer, a check of available information on HUDs on the website of the regional body was made. HUD information for two regions, Emilia-Romagna and Toscana, was extracted directly from the website of the regional body managing their HUDs. The remaining regional bodies were repeatably contacted until all accepted to participate in the survey. A summary of the survey procedure is shown Figure 1.

The following HUD characteristics were recorded: (i) name of the database, name of the database manager(s), start year and period covered, reference legislation; (ii) observation unit, type of information recorded, population covered, and size thereof; (iii) DMS, disease classification, type of personal identification code, and anonymization procedure used to protect patient identity (if any); (iv) missing values in specific fields and procedures and periodicity of data quality control; (v) data sources; (vi) frequency of data transmission from the sources to the regional/provincial administration.

The information obtained for each HUD was uploaded on the ARCHES project webpage [20], where it can be accessed from the region/province list or HUD list, by start year, by a combination of these criteria, or through a geographical map reporting the number of HUDs identified in each region/province.

A descriptive statistical analysis was performed to evaluate the main characteristics of the HUDs. Firstly, HUDs were grouped into three categories, 1—healthcare services, 2—conditions, diseases, other events, 3—other, based on the observational unit (e.g., healthcare service, beneficiary with a disease, any other events related to the health status or healthcare). In particular, category 1 included hospital discharge, outpatient care, residential care and hospice, home healthcare, mental healthcare, spa treatments, substance addiction treatment, blood transfusion services, cross-border healthcare, and emergency care; category 2 included disease registries, cancer registries, mortality registries, rare disease registries, infectious disease registries, accident registries, occupational health and safety registries, medical birth databases, spontaneous abortions databases, legal voluntary termination of pregnancy (VTP) databases, and screening registries; category 3 included NHS beneficiaries databases, co-pay exemptions databases, general practitioner registers, clinical laboratory services databases, drug dispensing databases (by healthcare facilities and hospitals and through contracted pharmacies), pathological anatomy databases, prosthesis registries, and vaccination registries.

Secondly, the absolute and percentage distributions of HUDs within each category were calculated according to region, start year, data management system, personal identification code, anonymization, coding system, data quality control, and data transmission.

## 3. Results

The survey identified 352 HUDs meeting the study criteria.

The geographical distribution of the HUDs in relation to the three categories is reported in Table 1. HUDs ranged from 39 in the province of Trento (Northern Italy) to six in Sardegna (southern Italy/Islands). Healthcare services databases were the most frequent and were found in nearly all regions.

*Start year.* The databases were set up from 1970 to 2016. The first was the mortality registry of Valle d’Aosta (northwest Italy) (Table S2, Supplementary Materials). Most HUDs (42%) were established after 2006, 22% were set up from 2000 to 2006, and 26% were set up before 2000; this information was not available for about 10% of HUDs (Table 2). As expected, mandatory HUDs were present in nearly all regions, although they were set up in different years (Table S2, Supplementary Materials). Some of the other HUDs were found only in three or fewer regions; in particular, a vaccination database was identified in three regions in northern Italy, a pathological anatomy database was identified in two regions in northern and central Italy, and a clinical laboratory services registry, an occupational health and safety registry, and a blood transfusion services and accident registry were identified in two regions in northern Italy.

*Data Management System (DMS).* Different DMSs were in use in the different regions and HUD categories (Table 3). Even though information on the software used was not available in almost 25% of HUDs, Oracle was the most common DMS in the healthcare services and conditions, diseases, other events categories, whereas Structured Query Language, SQL was the most common in the other databases. Other software (e.g., Java, Ippocrate, Netezza) was used in 14% of HUDs, and more than one DMS was employed in 11%. However, the same DMS was generally used for managing multiple HUDs within a region (Table S3, Supplementary Materials).

*Personal identification code*. Two personal identification codes were used in the HUDs: the unique identification code (ID) generated by regional authorities and the fiscal code (FC) (see Table 4 for the definitions). More than one type of identification code was used within each HUD category, as shown in Table 4; however, information was lacking for about one-fifth of HUDs. The unique ID was used by 46% of HUDs and the FC was used by around 26%; in 6% of HUDs, the personal identification code was not reported. To protect personally identifiable data, some regions did not use any ID in those HUDs that record highly sensitive information, such as data on spontaneous abortions, legal voluntary termination of pregnancy, and substance addiction treatment (3%, Table S4, Supplementary Materials).

*Anonymization.* The techniques used to anonymize personal data included separation (the procedure whereby any element that can lead to direct identification from personal data is removed and stored separately, with only a reference number left to allow re-identification by authorized parties), pseudonymization (an encrypted pseudonym derived from the personal data), an unspecified internal procedure, or encryption. Different anonymization techniques were identified within the HUDs categories among regions. Separation was the most common (Table 5), followed by an unspecified internal procedure. Pseudonymization and encryption were employed less frequently. The anonymization method was not reported in about 27% of HUDs. However, a single technique was used across each HUD category in each region (Table S5, Supplementary Materials).

*Coding system.* The coding systems used in the HUDs are reported in Table 6. Disease classification was most commonly performed according to the International Classification of Diseases (ICD); 9th revision, Clinical Modification (CM), ICD9 CM [21]), which is the method used at the national level. Some HUDs and regions used the ICD 10th revision (ICD10 CM) [22]; in particular, as requested by the Word Health Organization (WHO), it was the system employed in the mortality registries (Trento, Lombardia, Marche, Puglia and Calabria), the mental healthcare registries (Trento, Veneto, Lombardia, Friuli-Venezia-Giulia, Emilia-Romagna), and the cancer (Lombardia, Umbria), substance addiction treatment (Emilia-Romagna), and disease (Piemonte) registries.

In all drug dispensing databases, drugs were identified either by the Anatomical Therapeutic Chemical (ATC) classification [21] or by the Italian authorisation number (AIC) number [23] (see Table S6, Supplementary Materials, for the definitions).

The morphological, pathological, and clinical classification of malignant tumors was according to international codes (ICD-O 3, Systematized Nomenclature of Medicine (SNOMED), the classification system of malignant tumors, TNM) in all Regions.

Demographic data (e.g., province, region, and country of residence) were entered using the coding systems provided by the Italian National Institute of Statistics.

*Data quality control.* In about 84% of HUDs, the relevant regional/provincial administration stated that data completeness and quality were checked periodically (Table 7). In 40% of HUDs, data quality control was performed automatically at the time of recording by checking agreement between the data value and the pre-established data format and by controlling for any missing values in required fields. This method was more frequent in healthcare service HUDs. Data quality control was performed within a month or within 3–12 months from data acquisition in one-quarter and one-fifth of HUDs, respectively, whereas it was not performed in about 2% of HUDs, all belonging to the conditions, diseases, other events category; this information was not reported for 14% of HUDs.

In 55% of HUDs, the presence and frequency of missing data was not mentioned; in 25%, a statement reported that there were no missing data; in 12%, missing data ranged from 1% to 50%, whereas, in 8%, missing data were mentioned but not quantified (data not shown).

*Data transmission.* Data were transmitted from the healthcare providers to the administration at the time of recording in 11% of HUDs (Table 8), within 3–12 months in about 25%, and within one month of being recorded in most cases. The frequency of data transmission was not defined in 3% of HUDs and was not available in about 11%.

## 4. Discussion

In this survey, we aimed to create an inventory of the Italian regional HUDs, to describe them, and to develop a tool to navigate through them. The survey identified 352 electronic healthcare databases meeting the study criteria and described them in terms of start year, data type and completeness, data management system (DMS), quality control strategy, and data protection procedures in place. The inventory of the regional HUDs found in Italy’s 19 regions and two autonomous provinces is now available on a dedicated page of the ARCHES project website [20].

We found a widely different number of HUDs and start years in each region/province which reflects a highly different data availability.

The considerable homogeneity found within each region/province in important HUD features like the unique personal identification code, the anonymization technique, and the DMS adopted enable record linkage across HUDs.

Among regions, we found that the classification systems for diseases and drugs adopted were fairly homogeneous; the fact that some regions employed a more recent ICD revision highlights their greater promptness in implementing WHO recommendations. Since most administrative HUDs are regulated by national law, the same revision should be adopted everywhere.

Our survey highlighted different anonymization procedures employed by the various regions that can penalize clinical and epidemiological studies; for instance, it may hamper follow-up studies of patients with long-term, severe, or rare diseases, who are often treated at out-of-region specialist centers. In Italy, the healthcare services delivered to each resident—including those supplied by out-of-region centers—are paid for by the region where the patient resides. Although information about the services supplied by the other region are sent to the region of residence, it may not be transmitted all at the same time, resulting in temporary inconsistencies between databases that may cause the same query to yield different responses depending on the time it is submitted. To overcome these limitations, thus also enhancing the ability of HUDs to be used for nationwide healthcare monitoring and assessment, the National Unique Personal Code was established in 2016 [24] and adopted in 2017 by all regional HUDs. In doing so, Italy followed the example of other European countries, such as the Scandinavian countries, where a unique personal identification number assigned to permanent residents enables linkage of their records across multiple registries and databases [25,26,27].

The above results show the importance to explore the characteristics of different HUDs in a specific population and of the same types of HUDs in different populations and to provide a panel of metadata. The availability of these metadata facilitates the planning of epidemiological studies and regional and national studies to evaluate healthcare assistance. The difficulty to source information on HUDs characteristics could limit the studies based on large populations. In our survey, we found that data quality control procedures were in place in most HUDs but were characterized by widely different timing and methods both within and among regions. Data quality and completeness play a major role in supporting the validity of studies based on secondary sources and the ability of results to be generalized [28,29], especially because such studies are particularly prone to misclassification and the influence of confounding factors [11]. Addressing these problems requires application of appropriate study protocols and quality standards [30,31,32], as well as state-of-the-art methods of data analysis [7,8,33,34,35,36,37]. Awareness of the data quality control approach applied in a database can help users choose their data sources, design their study, and plan data analysis.

Some limitations should be considered in this survey. A selection bias may have occurred. We contacted the regional body managing HUDs, but different HUDs are often managed by different regional officials, who may not have all been contacted and involved in the survey by the manager of the regional HUDs. As a result, some HUDs may have been missed, explaining the small number of HUDs identified in some regions.

In some regions, the technical aspects of HUDs are managed by more than one person or by contractors, which may explain some missing responses on certain HUD features, like the anonymization procedure, software, and coding system used. To handle such information bias, after the survey results were uploaded on the ARCHES website [20], the managers of the regional HUDs were invited to check the data for inaccuracies or missing information.

The selection of the 38 HUDs with information from the regional website may have biased our results. The percentages of missing data regarding data management system, personal identification code, anonymization, and data quality control were significantly higher than the 314 HUDs selected by the administrated or self-administrated questionnaires (data not shown). No significant difference was observed between the latter two. However, the concerned HUDs were only 38 among the 352 identified HUDs, suggesting a negligible entity of the error.

Our survey brings some noteworthy strengths. To the best of our knowledge, it is the first study that gives insights into the activated HUDs and their types in each of the Italian regions, describing their main characteristics. It provides a tool for navigating through Italy’s regional HUDs, useful to researchers and those involved in healthcare evaluation to easily retrieve the information they require for epidemiological, clinical, and translational research and for healthcare system performance assessment. Our survey provides useful indication to identify the needed actions to optimize the use of HUD data. It employs an accurate methodology to identify HUDs, to describe them, to individuate their critical aspects, and to provide metadata to researchers, giving them the opportunity to know the information asset of a national situation in which the responsibility to manage health information is entrusted to the regions.

In practice, results of the survey may have important implications helping to fill the lacking knowledge of the activated HUDs in the Italian regions and their characteristics; therefore, they may enhance the use of real-world data in epidemiological and clinical research. On the other hand, the concerned health authorities, both local and national, could use the findings to improve homogeneity in and across regions, to provide updates, and to ensure that users can easily retrieve the information they require to foster the use of real-word data. In an international context, the survey and the tool for navigating HUDs could be a model to for other countries to provide information with regard to the available HUDs of their own country, covering a defined population and their features. The availability of metadata on the organization and the characteristics of HUDs could be important information to plan supranational projects using secondary sources of data. The possibility to retrieve these metadata in a standardized manner in different countries allows knowing and comparing the information asset between countries. In addition, these metadata could be helpful to avoid barriers for the protection of individuals with regard to the processing and circulation of personal data when HUDs are linked and used for epidemiological purposes, as already discussed by the Working Group on Observational Studies of the Italian Society of Medical Statistic and Clinical Epidemiology [38,39]. For these reasons, the proposed model could also be useful in the European context, which recently oriented health programs toward actions for a sustainable solid infrastructure on European health information through improving the availability of comparable, robust, and policy-relevant population health data and health system performance information [40]. Future updates of this dynamic tool are needed to ensure researchers and institutions with current information on the available HUDs.

## 5. Conclusions

In this survey, an inventory of the real-world regional databases of healthcare and related services managed by the regional/provincial administrations in Italy, as well as an examination of their characteristics, was provided. The ARCHES website represents a dynamic tool that allows researchers and institutions to get insights into the available Italian regional HUDs.

Results of our survey pointed out some critical issues that hamper the use of these secondary data sources in epidemiological studies, comparative effectiveness research, and assessments of healthcare system performance and health technology. Therefore, it highlights the need to improve homogeneity across regions that will allow improving the comparability of health data from secondary sources.

The survey and the tool for navigating HUDs proposed in this study can be considered as a useful model for other countries to provide information to researchers and institutions about the available HUDs in their countries. This will promote and enhance supranational large-scale epidemiological studies based on the use of health secondary data sources and will stimulate the sharing of standardized procedures to retrieve and compare the information asset between countries. Our study is consistent with the European strategy and activities on health information aiming to ensure better availability and use of health data for policy and research.

## Figures and Tables

**Table 1:** Regional healthcare utilization databases (HUDs) identified in Italy grouped by region and HUD category.

Geographical Area	Regions	Population Covered *		HUD Categories		Total
			Healthcare Services	Conditions, Diseases, Other Events	Other Databases	
North	Piemonte	4422	10 (37; 5.6)	11 (40.7; 11.7)	6 (22.2; 7.7)	27 (7.7)
	Valle d’Aosta	128	9 (42.9; 5)	7 (33.3; 7.4)	5 (23.8; 6.4)	21 (6)
	Liguria	1582	11 (73.3; 6.1)	1 (6.7; 1.1)	3 (20; 3.8)	15 (4.3)
	Lombardia	9995	6 (42.9; 3.3)	2 (14.3; 2.1)	6 (42.9; 7.7)	14 (4)
	Province of Trento	537	15 (38.5; 8.3)	17 (43.6; 18.1)	7 (17.9; 9)	39 (11.1)
	Province of Bolzano	518	6 (66.7; 3.3)	1 (11.1; 1.1)	2 (22.2; 2.6)	9 (2.6)
	Veneto	4923	12 (52.2; 6.7)	6 (26.1; 6.4)	5 (21.7; 6.4)	23 (6.5)
	Friuli Venezia Giulia	1226	7 (46.7; 3.9)	4 (26.7; 4.3)	4 (26.7; 5.1)	15 (4.3)
	Emilia-Romagna	4448	14 (77.8; 7.8)	2 (11.1; 2.1)	2 (11.1; 2.6)	18 (5.1)
Center	Marche	1549	11 (40.7; 6.1)	10 (37; 10.6)	6 (22.2; 7.7)	27 (7.7)
	Toscana	3749	10 (50; 5.6)	4 (20; 4.3)	6 (30; 7.7)	20 (5.7)
	Umbria	894	7 (58.3; 3.9)	3 (25; 3.2)	2 (16.7; 2.6)	12 (3.4)
	Lazio	5884	6 (50; 3.3)	4 (33.3; 4.3)	2 (16.7; 2.6)	12 (3.4)
South and Islands	Campania	5861	6 (66.7; 3.3)	-	3 (33.3; 3.8)	9 (2.6)
	Abruzzo	1331	7 (63.6; 3.9)	2 (18.2; 2.1)	2 (18.2; 2.6)	11 (3.1)
	Molise	313	4 (40; 2.2)	4 (40; 4.3)	2 (20; 2.6)	10 (2.8)
	Puglia	4086	4 (36.4; 2.2)	4 (36.4; 4.3)	3 (27.3; 3.8)	11 (3.1)
	Basilicata	576	15 (75; 8.3)	1 (5; 1.1)	4 (20; 5.1)	20 (5.7)
	Calabria	1976	5 (35.7; 2.8)	7 (50; 7.4)	2 (14.3; 2.6)	14 (4)
	Sardegna	1662	4 (66.7; 2.2)	1 (16.7; 1.1)	1 (16.7; 1.3)	6 (1.7)
	Sicilia	5087	11 (57.9; 6.1)	3 (15.8; 3.2)	5 (26.3; 6.4)	19 (5.4)
	Total	60,748	180 (51.1)	94 (26.7)	78 (22.2)	352 (100)

Unless otherwise indicated, data are reported as absolute frequency (row percentage; column percentage). * Mean of regional population, as thousands of residents between 2014 and 2016 according to the Italian National Institute of Statistics.

**Table 2:** HUD start year by HUD category.

HUD Categories		Start Year			Total
	<2000	2000–2006	≥2006	Not Reported	
Healthcare services	37 (19.9; 39.8)	31 (16.7; 40.3)	97 (52.2; 65.5)	21 (11.3; 61.8)	186 (52.8)
Conditions, diseases, other events	42 (44.7; 45.2)	28 (29.8; 36.4)	19 (20.2; 12.8)	5 (5.3; 14.7)	94 (26.7)
Other databases	14 (19.4; 15.1)	18 (25; 23.4)	32 (44.4; 21.6)	8 (11.1; 23.5)	72 (20.5)
Total	93 (26.4)	77 (21.9)	148 (42)	34 (9.7)	352 (100)

Data are reported as absolute frequency (row percentage; column percentage).

**Table 3:** Data management system (DMS) used in the HUDs according to HUD category.

DMS		HUD Categories		Total
	Healthcare Services	Conditions, Diseases, Other Events	Other Databases	
Oracle	37 (48.7; 20.6)	24 (31.6; 25.5)	15 (19.7; 19.2)	76 (21.6)
SQL	35 (53; 19.4)	11 (16.7; 11.7)	20 (30.3; 25.6)	66 (18.8)
SAS ^§^	22 (57.9; 12.2)	9 (23.7; 9.6)	7 (18.4; 9)	38 (10.8)
Other ^#^	17 (34; 9.4)	24 (48; 25.5)	9 (18; 11.5)	50 (14.2)
More than one	25 (62.5; 13.9)	5 (12.5; 5.3)	10 (25; 12.8)	40 (11.4)
Not reported	44 (53.7; 24.4)	21 (25.6; 22.3)	17 (20.7; 21.8)	82 (23.3)
Total	180 (51.1)	94 (26.7)	78 (22.2)	352 (100)

Data are reported as absolute frequency (row percentage; column percentage); ^§^ Statistical Analysis System ^#^ Java, Excel, Access, Sequential archive, Ippocrate, Netezza (a platform of the Ministry of Health), or not specified.

**Table 4:** Type of personal identification code used in the HUDs according to HUD category.

Type of Identification Code		HUD Categories		Total
	Healthcare Services	Conditions, Diseases, Other Events	Other Databases	
Unique identification code ^§^	90 (55.2; 50)	39 (23.9; 41.5)	34 (20.9; 43.6)	163 (46.3)
Fiscal code ^#^	49 (52.7; 27.2)	20 (21.5; 21.3)	24 (25.8; 30.8)	93 (26.4)
No code	38 (50.7; 21.1)	20 (26.7; 21.3)	17 (22.7; 21.8)	21 (6)
Not reported	3 (14.3; 1.7)	15 (71.4; 16)	3 (14.3; 3.8)	75 (21.3)
Total	180 (51.1)	94 (26.7)	78 (22.2)	352 (100)

Data are reported as absolute frequency (row percentage; column percentage). ^§^ Unique identification code: a sequential number assigned to the observation unit in the database according to the order of registration. ^#^ Fiscal code: in Italy, the tax code, which identifies each subject in their dealings with the public administration and in dealings between the public administration and other public or private entities. It consists of letters and numbers based on the subject’s first name, family name, and date and place of birth.

**Table 5:** Method used to anonymize personal data by HUD category.

Anonymization Method		HUD Categories		Total
	Healthcare Services	Conditions, Diseases, Other Events	Other Databases	
Separation	54 (64.3; 30)	15 (17.9; 16)	15 (17.9; 19.2)	84 (23.9)
Pseudonymization	16 (57.1; 8.9)	8 (28.6; 8.5)	4 (14.3; 5.1)	28 (8.0)
Internal procedure	38 (48.1; 21.1)	18 (22.8; 19.1)	23 (29.1; 29.5)	79 (22.4)
Encryption	15 (57.7; 8.3)	5 (19.2; 5.3)	6 (23.1; 7.7)	26 (7.4)
No anonymization	8 (20; 4.4)	24 (60; 25.5)	8 (20; 10.3)	40 (11.4)
Not reported	49 (51.6; 27.2)	24 (25.3; 25.5)	22 (23.2; 28.2)	95 (27.0)
Total	180 (51.1)	94 (26.7)	78 (22.2)	352 (100)

Data are reported as absolute frequency (row percentage; column percentage).

**Table 6:** Types of coding systems used in HUD categories.

Classification Systems		HUD Categories		Total
	Healthcare Services	Condition, Diseases, Other Events	Other Databases	
ICD9/10 CM	97 (64.2; 53.9)	48 (31.8; 51.1)	6 (4; 7.7)	151 (42.9)
ATC/AIC ^§^	0	0	21 (100; 26.9)	21 (6.0)
Other *	2 (10.5; 1.1)	9 (47.4; 9.6)	8 (42.1; 10.3)	19 (5.4)
No codes	4 (21.1; 2.2)	10 (52.6; 10.6)	5 (26.3; 6.4)	19 (5.4)
Not Reported	77 (54.2; 42.8)	27 (19; 28.7)	38 (26.8; 48.7)	142 (40.3)
Total	180 (51.1)	94 (26.7)	78 (22.2)	352 (100)

Data are reported as absolute frequency (row percentage; column percentage). ^§^ Anatomical Therapeutic Chemical classification/ Italian authorisation number for medicines; * Other includes (1) Italian National Institute of Statistics to code province, region, and country of residence and birth; (2) co-payment exemption code provided by the Italian Ministry of Health (Ministero della Salute (1999). Decreto 28 maggio 1999, n. 329. http://www.trovanorme.salute.gov.it/norme/dettaglioAtto?id=18796. Accessed on 11 March 2019); (3) Systematized Nomenclature of Medicine (SNOMED, https://www.snomed.org/ Accessed on 4 April 2019); (4) International Classification of Primary Care (ICPC, http://www.globalfamilydoctor.com/groups/WorkingParties/wicc.aspx. Accessed on 4 April 2019); (5) morphologic codes (International Classification of Diseases for Oncology, 3rd Edition, ICD-O 3, http://www.who.int/classifications/icd/adaptations/oncology/en/ Accessed on 19 February 2019); (6) the classification system of malignant tumors, TNM (https://www.uicc.org/resources/tnm. Accessed on 4 February 2019).

**Table 7:** Frequency of the data quality control in HUD categories.

Data Quality Control		HUD Categories		Total
	Healthcare Services	Condition, Diseases, Other Events	Other Databases	
At the time of data recording	80 (56.3; 44.4)	32 (22.5; 34)	30 (21.1; 38.5)	142 (40.3)
≤1 month	45 (52.9; 25)	13 (15.3; 13.8)	27 (31.8; 34.6)	85 (24.1)
3 ≤ months ≤ 12	37 (53.6; 20.6)	24 (34.8; 25.5)	8 (11.6; 10.3)	69 (19.6)
No control	0	6 (100; 6.4)	0	6 (1.7)
Not reported	18 (36; 10)	19 (38; 20.2)	13 (26; 16.7)	50 (14.2)
Total	180 (51.1)	94 (26.7)	78 (22.2)	352 (100)

Data are reported as absolute frequency (row percentage; column percentage).

**Table 8:** Frequency of data transmission in the HUD categories.

Data Transmission		HUD Categories		Total
	Healthcare Services	Condition, Diseases, Other Events	Other Databases	
At the time of data recording	8 (21.1; 4.4)	23 (60.5; 24.5)	7 (18.4; 9)	38 (10.8)
<1 month	95 (55.2; 52.8)	25 (14.5; 26.6)	52 (30.2; 66.7)	172 (48.9)
3 ≤ months ≤ 12	52 (57.8; 28.9)	29 (32.2; 30.9)	9 (10; 11.5)	90 (25.6)
Not defined	5 (41.7; 2.8)	6 (50; 6.4)	1 (8.3; 1.3)	12 (3.4)
Not reported	20 (50; 11.1)	11 (27.5; 11.7)	9 (22.5; 11.5)	40 (11.4)
Total	180 (51.1)	94 (26.7)	78 (22.2)	352 (100)

Data are reported as absolute frequency (row percentage; column percentage).

## Supplementary Materials

The following are available online at https://www.mdpi.com/1660-4601/17/1/8/s1: Table S1: Questionnaire used to survey the HUDs covering the population of a single region/province and recording local-level data in Italy (self-administered or administered by one of the authors); Table S2: HUD start year according to region and category; Table S3: Type of data management system used in HUDs according to region; Table S4: Type of personal identification data used in HUDs according to the region; Table S5: The type of anonymization of the personal identification data used in HUDs according to the region; Table S6: Coding systems.
