# Supplementary material for: Availability of Real-World Data in Italy: A Tool to Navigate Regional Healthcare Utilization Databases
Source: Int J Environ Res Public Health. 2019 Dec 18;17(1):8. doi: 10.3390/ijerph17010008 (PMC6982131; doi:10.3390/ijerph17010008)
Supplement: Supplementary file 1 [file ijerph-17-00008-s001.docx]

**Table S1.** Questionnaire used to survey the HUDs covering the population of a single Region/Province and recording local-level data in Italy (self-administered or administered by one of the authors).

|  | **Compiler**  **[Family name, first name, phone, e-mail]** |  |
| --- | --- | --- |
|  | **Date of compilation** |  |
|  | **Region / Province / local NHS** |  |
| 1 | **Database name** |  |
| 2 | **Database managing body** |  |
|  | [Link] |  |
| 3 | **Database manager** [Family name, first name, phone] |  |
|  | [e-mail] |  |
| 4 | **Start date/activation** |  |
| 5 | Period of activity  [start date – end date] |  |
| 6 | **Notes** [suspension periods] |  |
| 7 | **Legal references** |  |
|  | [Link] |  |
| 8 | **Aim/Function** |  |
| 9 | **Type of information** |  |
| 10 | **Reference population** |  |
| 11 | **Population at the time of compilation** |  |
| 12 | **Notes** |  |
| 13 | **Database Management System** |  |
| 14 | **Observation unit** |  |
| 15 | **International disease code** |  |
| 16 | **Year code was changed / name of new code** |  |
| 17 | **Personal identification code** |  |
| 18 | **Anonymization of personal identification code?** |  |
| 19 | **Yes, by procedure** |  |
| 20 | **Missing data** [% of total records of the year preceding the compilation of the questionnaire]: |  |
| 21 | **- identification code** |  |
| 22 | **- biographical data** |  |
| 23 | **- specific fields** [1]  **- specific fields** [2] |  |
| 24 | **Notes** |  |
| 25 | **Data quality control** |  |
| 26 | **Control frequency** |  |
| 27 | **Data source / origin** |  |
| 28 | **Frequency of data transmission to the database managing body** |  |
| 29 | **Mode of data transmission to the database managing body** |  |
| 30 | **Database Management System**  [used by the data source/ origin] |  |

**Table S2.** HUD start year according to Region and category.

Regions: PIEM: Piemonte, VA: Valle d'Aosta, LIG: Liguria, LOMB: Lombardia, PA.T: Province of Trento, PA.B: Province of Bolzano, VEN: Veneto, FVG: Friuli Venezia Giulia, ER: Emilia-Romagna, MAR: Marche, TOSC: Toscana, UMB: Umbria, LAZ: Lazio, CAM: Campania, ABR: Abruzzo, MOL: Molise, PUGL: Puglia, BAS: Basilicata, CAL: Calabria, SARD: Sardegna, SIC: Sicilia.

NR: Data Not Reported. In grey HUDs mandatory for law and registered in national databases.

**Table S3.** Type of data management system used in HUDs according to Region.

Regions: PIEM: Piemonte, VA: Valle d'Aosta, LIG: Liguria, LOMB: Lombardia, PA.T: Province of Trento, PA.B: Province of Bolzano, VEN: Veneto, FVG: Friuli Venezia Giulia, ER: Emilia-Romagna, MAR: Marche, TOSC: Toscana, UMB: Umbria, LAZ: Lazio, CAM: Campania, ABR: Abruzzo, MOL: Molise, PUGL: Puglia, BAS: Basilicata, CAL: Calabria, SARD: Sardegna, SIC: Sicilia.

NR: Not Reported; More: More than one; Other: Java, excel, access, Sequential archive, Ippocrate, netezza, a platform of the Ministry of Health, not specified

**Table S4.** Type of Personal Identification data used in HUDs according to the Region.

Regions: PIEM: Piemonte, VA: Valle d'Aosta, LIG: Liguria, LOMB: Lombardia, PA.T: Province of Trento, PA.B: Province of Bolzano, VEN: Veneto, FVG: Friuli Venezia Giulia, ER: Emilia-Romagna, MAR: Marche, TOSC: Toscana, UMB: Umbria, LAZ: Lazio, CAM: Campania, ABR: Abruzzo, MOL: Molise, PUGL: Puglia, BAS: Basilicata, CAL: Calabria, SARD: Sardegna, SIC: Sicilia.

ID: Unique Identification code; FC: Fiscal Code; No: No Identification Code; NR: Not Reported

**Table S5.** The type of anonymization of the personal identification data used in HUDs according to the Region.

Regions: PIEM: Piemonte, VA: Valle d'Aosta, LIG: Liguria, LOMB: Lombardia, PA.T: Province of Trento, PA.B: Province of Bolzano, VEN: Veneto, FVG: Friuli Venezia Giulia, ER: Emilia-Romagna, MAR: Marche, TOSC: Toscana, UMB: Umbria, LAZ: Lazio, CAM: Campania, ABR: Abruzzo, MOL: Molise, PUGL: Puglia, BAS: Basilicata, CAL: Calabria, SARD: Sardegna, SIC: Sicilia.

Encr: Encryption; Int: Internal procedure; Pseud: Pseudonymization; Sep: Separation, No: No Anonymization; NR: Not Reported.

**Table S6.** Coding Systems.

| **Coding systems** | **Description** | **Link^*^** |
| --- | --- | --- |
| ICD9/10 | International Classification of Disease, 9^th^ revision, Clinical Modification or 10th revision | <https://www.cdc.gov/nchs/icd/index.htm> |
| ATC/AIC | Anatomical Therapeutic Chemical classification system, or Authorization number issued by the Italian Medicines Agency AIFA | [https://farmaci.agenziafarmaco.gov.it/bancadatifarmaci/cerca-farmaco; https://www.whocc.no/atc_ddd_index/](https://www.whocc.no/atc_ddd_index/) |
| ISTAT | Italian National Institute of Statistics to code province, region and country of residence and birth | https://www.istat.it/it/archivio/6789 |
| Exemptions | Co-payment exemption code provided by the Italian Ministry of Health (Ministero della Salute,1999) | [Decreto 28 maggio 1999, n. 329. http://www.trovanorme.salute.gov.it/norme/dettaglioAtto?id=18796)](http://www.trovanorme.salute.gov.it/norme/dettaglioAtto?id=18796) |
| SNOMED | Systematized Nomenclature of Medicine | <https://www.snomed.org/> |
| ICPC | International Classification of Primary Care | <http://www.globalfamilydoctor.com/groups/WorkingParties/wicc.aspx> |
| ICD-O3 | Morphologic codes | <http://www.who.int/classifications/icd/adaptations/oncology/en/> |
| TNM | Classification of Malignant Tumors | <https://www.uicc.org/resources/tnm> |

^*^ Accessed on February 21, 2019 ^.^
